# Nudging Finnish Adults into Replacing Red Meat with Plant-Based Protein via Presenting Foods as Dish of the Day and Altering the Dish Sequence

**DOI:** 10.3390/nu14193973

**Published:** 2022-09-24

**Authors:** Esa-Pekka Nykänen, Ulla Hoppu, Eliisa Löyttyniemi, Mari Sandell

**Affiliations:** 1 Functional Foods Forum, University of Turku, 20014 Turku, Finland; 2 Department of Biostatistics, University of Turku, 20520 Turku, Finland; 3 Department of Food and Nutrition, University of Helsinki, 00014 Helsingin Yliopisto, Finland

**Keywords:** food choice, nudging, dish of the day, sequence alteration, buffet meal, plant-based protein

## Abstract

This study investigated whether Finnish working-aged omnivores (n = 163) could be nudged into replacing red meat with a fava-bean-based protein source via “Dish of the Day” (DoD) and main dish sequence alteration (SA) strategies in a controlled real-world Finnish self-service buffet restaurant with smart scales (Flavoria^®^ Multidisciplinary Research Platform). A further aim was to study whether the effectiveness of the strategies differed by gender, age, and body mass index. The participants were assigned one of four experimental treatments: standard menu (T1), DoD (T2), standard menu + SA (T3), or DoD + SA (T4). The participants could choose any amount or combination of salad components and casseroles with minced meat or fava bean protein. Being subjected to a DoD menu and/or SA had no effect on main dish choice or the share of the meat-based dish in the meal weight. Men were more likely to choose a meat-based main dish and had a higher share of the meat dish in the meal weight compared to women, but no differences were observed between those aged 18–29, 30–44, or 45–65 years or those who were normal weight, overweight, or obese. Future studies should have a larger sample size and investigate food choice motives such as price or environmental awareness.

## 1. Introduction

The latest report from the Intergovernmental Panel on Climate Change (IPCC) [1] concluded that global warming has continued at an even faster pace than that previously projected. Immediate action is required in order to avoid substantial further damage caused by global warming [1].

Food production and consumption are major drivers of climate change [2,3]. The EAT Lancet Commission concluded that there is a strong need to reduce the consumption of animal-based foods and increase the consumption of plant-based foods globally for both environmental and health reasons [4].

One promising tool to enhance more sustainable and healthy food choices is nudging [5]. The concept of nudging was originally presented by Thaler & Sunstein [6] and it is based on an idea that factors such as anchoring, framing, and status quo bias have an effect on human decision making. Therefore, so-called “choice architecture” may have an impact on the final decision [6]. Choice architecture refers to not restricting an individual’s choices but instead making changes to the environment where the decisions are made [5]. A systematic review of reviews conducted by Wright and Bragge [7] concluded that nudging strategies focusing on social norms and modeling can have a strong influence on human food consumption.

One approach leveraging social norms in order to influence a consumer’s food choices has been to change the default options in different food service settings [8,9]. A randomized controlled field experiment from Hansen et al. [8] suggested that merely presenting a plant-based meal option as a default option in an invitation letter may significantly increase the likelihood of choosing it instead of an animal-based meal option among the adult population. Campbel-Arvai et al. [9] found that configuring menus and presenting meat-free options as default options increased the likelihood of university students choosing them in a university campus setting.

The so-called “Dish of the Day” (DoD) strategy has been tested in various settings [9,10,11,12]. In terms of factors influencing the effectiveness of DoD strategies, Saulais et al. [13] concluded that the effect of the DoD strategy was higher for unpopular dishes and when multiple dish options were served. Hartwell et al. [11] found that combining both creative menu and meal designing were particularly effective in their food-service setting. Regarding the effectiveness among different age groups, the DoD strategy was effective in influencing dish selection among adolescent females but not among males or older people [11]. However, presenting a plant-based meal option as a DoD did not produce the same effect among European adolescents [10] or those above the age of 65 years in Europe [12].

Review articles by Hollands et al. [14] and Bucher et al. [15] suggest that the sequence in which the foods are presented in the physical environment may also play a role in the food selection. Hollands et al. conclude that more research needs to be conducted in real-life settings in order to obtain more solid conclusions [14]. Furthermore, Bucher et al. concluded that the outcomes should be measured on a gram-level change in food consumption in order to measure the full effect [15].

As suggested by Dos Santos et al., nudging strategies may not work equally well in all cultural contexts [10]. According to the latest national dietary intake study, *FinRavinto* 2017, Finnish people’s diets contain high amounts of animal-based products and less than recommended amount of vegetables [16]. In total, 79% of men and 26% of women consumed more red or processed meat than the current maximum recommended amount of 500 g per week [16]. The Finnish Environment Institute [17] has recommended reducing the consumption of ruminant meat as one of the key action points in order to reduce Finnish people’s personal carbon footprints. Excessive red meat consumption also has direct negative health consequences for the Finnish population as it has been associated with increased risks of developing colorectal cancer, obesity, type-2 diabetes, and coronary heart disease [18]. 

Welfare policies ensuring access to reasonably priced and nutritious meals during workdays have been implemented in Finland [19]. Prior to the COVID-19 pandemic, approximately one-third of working-aged Finnish people ate lunch in staff canteens on a daily basis [16]. In cases where a staff canteen was available for the employees, it was the most common choice for lunch for both men and women (38% and 43%, respectively) [16]. Typically, in Finland, salad components are offered as part of lunch meals in staff canteens. Due to the high volume of customers [16], staff canteens can be considered as good settings for promoting more environmentally friendly eating habits.

Previous studies conducted in Finland have looked at the implementation and feasibility of choice architecture cueing [20], but to the best of the authors’ knowledge, the effect of the DoD and SA strategies have not been studied among the Finnish adult population. Few studies [21,22] have investigated the effect of sequence alteration (SA) in a buffet setting, which is a common context in which Finnish adults have their lunch [16]. In order to address this, this study was conducted in a real-world Finnish lunch restaurant (Flavoria^®^ Multidisciplinary Research Platform) [23]. Furthermore, the food choices were measured with smart scales providing gram-level data, as suggested by Bucher et al. [15].

The main goal of this study was to investigate whether Finnish working-aged omnivores could be nudged into choosing plant-based protein sources instead of red meat via a DoD strategy and altering the sequence of the main dishes in a real-world self-service buffet restaurant setting.

As suggested by the results of previous studies [11,12,24], personal factors such as sex, age, and body mass index (BMI) may play a role in the effectivity of the nudging strategies. Specifically, females [11,12], those with younger age [11], and those with high BMI [24] have been reported to be more likely to change their food choices after being subjected to nudging. Since the consumption of red meat differs by gender, age and BMI [16], there is a specific interest in identifying whether the nudging strategies are equally effective for different population subgroups. Therefore, the secondary aim was to gain knowledge of whether gender, age, and BMI had an impact on the effectiveness of the DoD and SA strategies in a Finnish context.

## 2. Materials and Methods

### 2.1. Participants

Finnish-speaking persons aged 18–65 whose diet contained meat and who did not have food allergies or intolerances were invited to participate in the study. Participants were recruited using several channels such as the Functional Foods Forum’s consumer register, advertisements at the restaurant and on Facebook, push notifications in the MyFlavoria^®^ mobile application, and via an email list of employees working in the same building where the Flavoria^®^ Multidisciplinary Research Platform is located. University of Turku (UTU) channels such as the web page, weekly newsletter for employees, and email lists of student organizations were also used in the recruitment process.

During the data collection period between October and December 2021, COVID-19 was highly present in Finland, and the public recommendation to work remotely was active, which hindered the recruitment of participants. Many last-minute cancellations were expected due to the high load of cases and people that had to quarantine. This made predictions about the final turn-out percentage very difficult. Therefore, in order to obtain a sufficient sample size, potential participants were given the possibility to choose between several time slots to participate in the study based on their personal preferences. In total, a random sample of 163 participants was collected. 

The participants gave consent for the use of their data for research purposes while signing up to the study via an online registration form (Supplementary File S1). The UTU COVID-19 safety protocol was closely followed during the data collection process: people were advised to maintain safe distances and wear face masks, which, along with hand sanitizers and hand washing stations, were made available to the participants before and after the participation. All staff members were wearing face masks during the data collection process.

The Flavoria^®^ study protocol was reviewed and ethically approved by the Ethics Committee for Human Sciences at the UTU, Humanities and Social Sciences Division (37/2021). The study followed the European Union’s General Data Protection Regulation (GDPR).

### 2.2. Food Components Served in the Study Buffet

**Figure 1:**
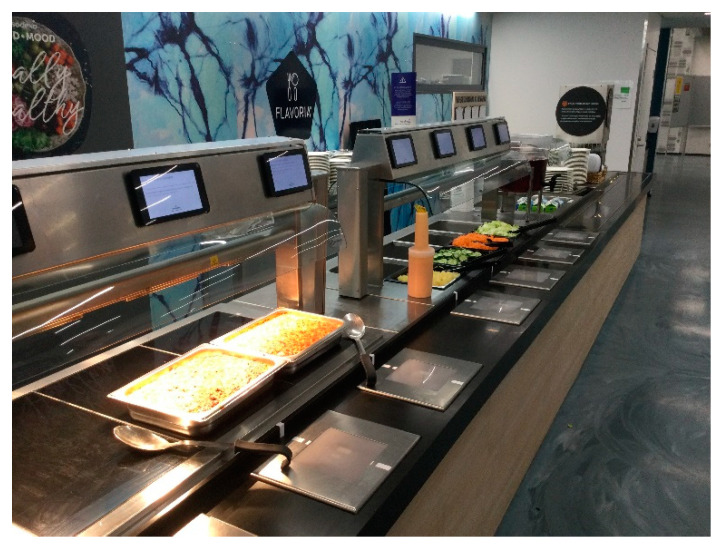
View of the buffet line.

A buffet-style meal with salad, bread, and drinks (water, milk, and/or juice) was offered at the Flavoria^®^ Multidisciplinary Research Platform, Turku, Finland [23], which is where the food service is operated by the Sodexo company [25]. The participants were able to choose an unlimited amount of iceberg lettuce, shredded carrots, cucumber slices, canned pineapple bits, fava bean casserole (FBC), and minced meat casserole (MMC) from the buffet line with integrated scales (see Figure 1). MMC is a traditional Finnish dish. It was prepared from pasta, minced beef, diced onions, meat bouillon, eggs, cow’s milk, iodized salt, black pepper, and ground dried bell pepper. The FBC was prepared from the same ingredients except that the bouillon was replaced with vegetable stock and minced beef with Härkis^®^, which is a plant-based protein-rich product resembling minced meat. It is made from 52% fava beans, water, pea protein, canola oil, 0.8% of iodized salt, syrup, modified starch, sugar color, stabilizers E460 and E461, plant fiber, onion extract, and black pepper [26]. Both of the dishes were identical in terms of appearance (see Supplementary File S2).

Additional trimming components such as 1.5% fat cow’s milk, 0.1% fat cow’s milk, soured milk, soy milk, mixed berry juice, white, dark, and crisp bread, butter, margarine, olive oil, salad dressing, and ketchup were also served, and their intakes were measured using a questionnaire.

The served meal with coffee and/or tea and a choice of small chocolate bar as a dessert were offered for free to the participants as compensation for their time. No other rewards were given.

### 2.3. The Nudging Strategy

**Figure 2:**
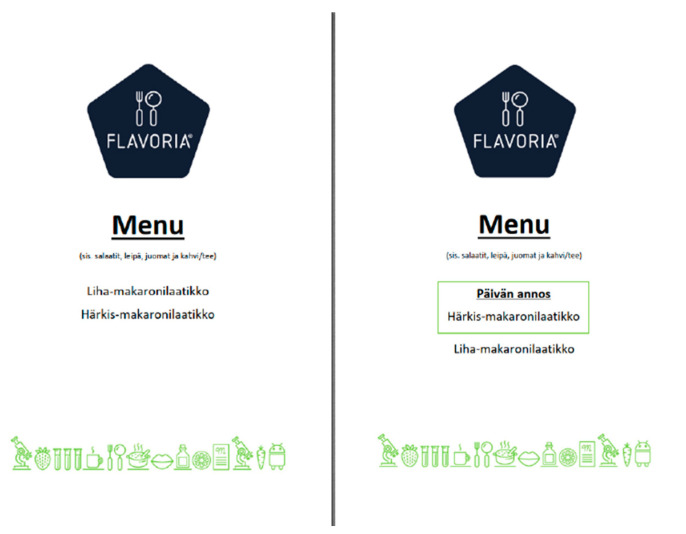
The standard menu (**left**) and Dish of the Day (*in Finnish “Päivän annos”*) (**right**) menu. The Finnish text “*sis. salaatit, leipä, juomat ja kahvi/tee*” can be translated as “including salads, bread, drinks, and coffee/tea” into English. The Finnish words *Liha-makaronilaatikko* and *Härkis-makaronilaatikko* refer to minced meat casserole and fava bean casserole.

At the moment of arrival, the participants were presented either a standard menu (in which the MMC was presented above the FBC, see Figure 2, left) or a DoD menu (see Figure 2, right) in which the FBC (in Finnish *Härkis-makaronilaatikko*) was surrounded with a light green frame and a text “Päivän annos” (in English, *Dish of the Day*).

The participants were registered into one of the four experimental treatments depending on the moment of arrival. The first group (T1) received a standard menu (see Figure 2, left), and the MMC was served before the FBC at the buffet line at the moment of entry. The second group (T2) received a DoD menu (see Figure 2, right), while the MMC was also served first. The third group (T3) received a standard menu, while the FBC was served before the meat version (see Figure 1, right). The fourth group (T4) received a DoD menu, while the FBC was served before the meat version.

On the first study day, the participants who arrived first were assigned into T1, and those who came after were assigned into T2, T3, and T4, respectively. On the second day, the order was T2, T3, T4, and T1; on the third day, it was T3, T4, T1, and T2, and on the fourth day, it was T4, T1, T2, T3. The research staff collected menus from each participant after the registration to avoid potential contamination bias. For the same reason, the sequence of the main dishes in the buffet line was changed, while the participants were waiting in a nearby room.

### 2.4. The Flavoria^®^ Multidisciplinary Research Platform Self-Service Restaurant Setting

The data were collected on four days between October and December 2021: on the first day between 4 PM and 7 PM and on the other three days between 3:30 PM and 6:30 PM. The self-serve buffet line had scales in front of each dish (see Figure 1), and the amount of food taken by each participant was registered into a server [27]. When the tray was set on the first scale a corresponding session ID was created, and the following measurements were registered under the same session ID. A time stamp of each measurement was recorded under each session ID.

Each dish/food had a corresponding screen on top of it (see Figure 1), which contained the name and allergens of each food as well as the following information: changing symbols telling the participant when the scale was being calibrated (an hourglass symbol) and when the participant could start taking food on the plate (a ladle symbol), the amount of food taken (grams), and when the measurement was completed (a green tick). More detailed information regarding the technical aspects of the Flavoria^®^ Multidisciplinary Research Platform have been published and are available elsewhere [27].

### 2.5. Procedure

The participants were given individual ID numbers at the start of the study. Each tray had a corresponding ID sticker on the left upside corner of each tray. The participants were instructed by the research staff on how to use the scales correctly before taking food and were observed and re-instructed while taking food. The staff recorded scaling errors (n = 8), which were manually corrected into the dataset afterwards. A photo of the meal was taken by the research staff, and in case of the missing data (one main dish and one meal with three meal components), the correct food items were identified from the photos and verified from the questionnaire answers. The missing values were replaced with the day’s mean portion sizes of each dish or food item.

After the meal, the participants filled out an online questionnaire (Webropol Version 3.0) [28] designed by the research team (the original questionnaire in Finnish) using Apple iPads or with their smartphones by scanning a QR-code. The main themes of the questionnaire were the participant’s background (e.g., gender, birth year, height, and weight), familiarity with Härkis^®^, and curiosity towards new foods. Individual ID numbers given at the start of the study were requested and were used to match the questionnaire data with the meal data.

One participant from T1 was unable to respond to the questionnaire due to a technical error. This participant was excluded from the part of the analysis requiring the data collected via the questionnaire (e.g., gender or age).

### 2.6. Statistical Analyses

Continuous variables are presented with means and standard deviation (SD) and categorical variables with counts and proportions (%). The results of the model are presented with model-based means together with 95% confidence intervals (CI).

Fisher’s Exact tests (using Monte Carlo estimates) were used to study the differences in the categorical variables between the treatments, age was compared with the Kruskal–Wallis tests, and the logarithmic transformed BMI was analyzed using one-way analysis of variance.

The main dish choice was compared between the treatments using ordinal logistic regression, first with a univariate approach (having only treatment in the model) and then adjusted with gender, age group (18–29, 30–44, and 45–65), and BMI group (18.5–24.9, 25–29.9, and 30). Participants who either did not answer the questions regarding weight, height, or gender, or answered “Other” to gender were removed from the analyses.

The mean meal weight, mean amount of salad, and the share of the meat dish in the meal weight were analyzed using linear models, including categorical factors treatment, main dish choice, gender, age group and BMI group. Assumptions for linear models were checked using studentized residuals.

The data analysis for this paper was generated using SAS software, Version 9.4 of the SAS System for Windows.

## 3. Results

A total of 163 participants were divided into four treatments. Their ages ranged from 18 to 65 years, and the majority were women (59.5%) (Table 1). There were no significant differences between the treatments in terms of the gender distribution (men or women), mean age, BMI, frequency of consuming meat or plant-based proteins during the main meal of the day, frequency of trying new ingredients, or previous experience with Härkis^®^ (Table 1). In terms of employment status, there was a significant imbalance between the treatments (*p* = 0.048) (Table 1).

Table 2 presents the main course selection, mean meal weight, mean salad share, and main course share between the treatments. Gender differences in the crude meal weights and shares of the FBC and the MMC in the meal weight were found (Table 2).

### 3.1. Main Dish Choice

There were no statistically significant differences in the main dish choices between the treatments before (*p* = 0.13) or after adjusting for confounders (*p* = 0.23) (Table 3). After adjusting for treatment, BMI group, and age group, women were approximately 2.8-fold more likely to choose only FBC compared to men (OR: 2.77, 95% CI: 1.46, 5.26, *p* = 0.002). The main dish choices did not differ significantly between the different age groups before or after adjusting for confounders (Table 3).

There was a trend towards those with a higher BMI having a higher likelihood of choosing only MMC (*p* = 0.056) (Table 3). While there were no relevant differences in the likelihood of choosing only MMC between those who were normal weight and those who were overweight (33.8% and 35.4%, respectively), obese participants were more likely to choose only MMC compared to 33.8% of those with normal weight (51.8% and 33.8%, respectively).

### 3.2. Share of Minced Meat Casserole in the Total Meal Weight

There were no statistically significant differences in the mean share of the MMC in the total meal weight between the treatments when including all participants in the analysis (Table 4) (0.47, 0.36, 0.46, 0.39, respectively, *p* = 0.18). After adjusting for confounders, the differences remained non-significant (*p* = 0.33) (Table 4).

The multivariable model showed that the mean share of the MMC in the total weight was significantly higher among men compared to women (*p* < 0.0001). No statistically significant differences in the mean shares of the MMC were found between the age (*p* = 0.70) or BMI groups (*p* = 0.11).

### 3.3. Meal Weights 

After adjusting for confounders [(gender, age group, BMI group, and main dish choice (those who chose only FBC, only MMC, or chose both)], there were no statistically significant differences in the mean total meal weights between the treatments [T1: 445 g (95%CI: 404 g, 486 g), T2: 423 g (95%CI: 384 g, 463 g), T3: 413 g (95%CI: 371 g, 454 g), and T4: 447 g (95%CI: 403 g, 491 g), *p* = 0.56]. There was a statistically significant difference in the mean total meal weights between men and women [489 g (95%CI: 454, 525 g) and 375 g (95%CI: 349 g, 401 g), *p* < 0.001].

The mean meal weight was significantly higher among those aged 18–29 years [469 g (95%CI: 434 g, 504 g)] compared to the 46–65-year-olds [389 g (95%CI: 352 g, 427g)] (*p* = 0.005). No significant differences were observed when comparing the mean meal weights of the 30–45-year-olds [437 g, (95%CI: 399 g, 475 g)] to the other age groups (*p* = 0.39 and *p* = 0.14, respectively).

There were no statistically significant differences in the mean total meal weights between those who chose only FBC as the main dish [416 g, (95%CI: 368 g, 465 g)], those who chose only MMC [431 g, (95%CI: 398 g, 463 g)], or those who chose both [449 g, (95%CI: 417 g, 481 g), *p* = 0.50)].

The mean total meal weights did not differ significantly between those who were normal weight [422 g, (95%CI: 394 g, 451 g)], overweight [424 g, (95%CI: 388 g, 460 g)], or obese [449 g, (95%CI:401 g, 497 g), *p* = 0.60].

The amount of salad did not differ statistically between the treatments (*p* = 0.57), gender (*p* = 0.14), age groups (*p* = 0.43), BMI groups (*p* = 0.064), or three main dish choice groups (*p* = 0.50) (those who chose only FBC, only MMC, or both). 

## 4. Discussion 

### 4.1. Impact of Dish of the Day Strategy

In this study, nudging the participants via a DoD strategy and/or SA did not significantly increase Finnish adults’ likelihood to choose the FBC over the MMC in a real-life Finnish restaurant setting (Table 3). The results of previous research on the effectiveness of the DoD strategy have been mixed and seemingly dependent on context; Campbell-Arvai et al. found that presenting the meat-free options as default significantly increased the likelihood of choosing them compared to those who received a nondefault menu (odds ratio = 4.1) in an American university campus setting [9]. Saulais et al. found that presenting a chosen vegetarian meal as a DoD increased the likelihood of choosing it from 25.2% to 59.6% when having one vegetarian option to choose from and from 23.3% to 53.3% when another vegetarian meal was also available to restaurant customers with mean age of 51.6 years [13]. Hartwell et al. found that their DoD strategy increased adolescent females’ likelihood of selecting the plant-based dish, whereas no effect was observed among males or older people [11]. Similarly, Zhou et al. observed that a DoD strategy was not effective in increasing older Europeans’ likelihood of choosing a plant-based dish; however, the likelihood was higher among female participants compared to males and among those from the UK and Denmark compared to those from France [12]. The DoD strategy did not have an effect on European adolescents’ likelihood of choosing a plant-based dish [10]. 

No studies investigating the effect of DoD strategy among Finnish adults have been published prior to this study. The effectivity of the DoD strategy did not differ by the participants’ gender, age or BMI (Table 3). Therefore, it may be possible that the Finnish cultural context may have played a role in the effectivity of the DoD strategy.

### 4.2. Impact of Sequence Alteration of the Main Dishes

Changing the sequence of the main dishes in this study did not have an effect on the main dish choice, share of the meat dish in the meal weight, or amount of salad, compared to those participants who faced the meat option first (Table 3 and Table 4). Previously, a Cochrane review by Hollands et al. concluded that there was a very low certainty evidence of altering the sequence of dishes resulting in a reduction in their consumption [14]. Despite some studies conducted in buffets [21,22] or salad bars [29] finding evidence suggesting that SA may influence food choices, they cannot be compared to this study in terms of study design and cultural context: for example, Wansink and Hanks [22] investigated the effect of placing the foods in a sequence from healthiest to least healthy or vice versa in an American health conference buffet setting, and Kongsbak et al. [21] altered the serving sequence of salad components and other dishes in a Danish buffet setting. Rozin and colleagues [29] placed salad components in a more accessible position at the edge of the salad bar compared to having them placed in a less accessible position in middle of a two-sided buffet line. In this study, the main dishes were placed next to each other (see Figure 1), and the participant was already aware what was next in the buffet line due to seeing a menu beforehand. In this study, the main dishes were also very similar (see Supplementary File S2). In normal Finnish buffet restaurants, the salad components are already served before the main dish; therefore, only minor changes in food sequence were tested. Thus, direct comparison with other studies is not possible. The results may have been slightly different if the participants had come into the restaurant without having seen a menu and, therefore, were unaware of the food that was going to be served.

Despite Kongsbak et al., Wansink and Hanks and Rozin and colleagues’ [21,22,29] findings that SA may affect food choices, the differences in their findings may be simply due to more radical changes made to the sequence compared to the ones made in this study. Therefore, it is important to note that not all SA may be effective in promoting healthier food choices. The changes may need to be large enough to cause an impact and there may be cultural differences in the rate of effectivity of SA strategies. 

In this study, gender, age and BMI did not influence the effectivity of the SA (Table 3 and Table 4), which suggests that other behavior change strategies may prove to be more effective in targeting different population sub-groups in Finland.

### 4.3. Differences in Food Choices by Gender and Body Mass Index 

The share of the MMC in the total meal weight was compared in order to control for differences in the gender distribution and mean age between the treatments (Table 1). DoD and SA did not cause significant changes in the share of the MMC in the total meal weight (Table 4).

After controlling for treatment, age, and BMI group, men were more likely to both choose the MMC (Table 3) and have a larger share of the MMC in the total meal weight compared to women (Table 4). Several factors may explain the differences between men and women: first, Finnish women may be more likely to eat plant-based meat alternatives compared to Finnish men [30]. Second, biological differences in body size and in energy consumption might explain why men might choose more energy-dense meals. Another explanation could be that men might have connotations such as power with meat, and meat eating may be seen as more masculine [31] compared to eating plant-based meals. Women might also be more interested in health [32], which might be explain the larger share of salad in the meal weight.

A trend of those with a higher BMI being more likely to choose only MMC compared to those with a lower BMI was observed (Table 3). Niva and Vainio [33] have previously reported that health and sustainability motives have been associated with higher use of the plant-based alternatives in Finland. Those with a normal weight might, therefore, be more likely to choose vegetarian options due to stronger health motives. Interestingly when looking at the share of the MMC in the total meal weight, the trend was not statistically significant (Table 4). It has been observed that those who follow a diet characterized by a high intake of meat might be more likely to have a high BMI compared to those characterized by, e.g., a high intake of vegetables, beans, or fruits [34].

### 4.4. Strengths of the Study

To the best of the knowledge of the authors, this study was the first recorded attempt to investigate the effectivity of DoD and SA strategies in a Finnish context. Buffet restaurants are very common in Finland especially during the lunch hours [16]; therefore, the results can be generalized to other buffet restaurant settings in Finland. Compared to other studies investigating the effect of SA in a buffet settings where large changes in food sequence were studied [21,22], the minor changes implemented into the dish sequence in this study resembled a real-world Finnish buffet restaurant context.

Previously, Hollands et al. and Bucher et al. [14,15] have emphasized the importance of conducting more studies in real-world settings and highlighted the importance of measuring the food choices on a gram-level [15]. This study was conducted in a real-life Finnish buffet restaurant setting (Flavoria^®^ Multidisciplinary Research Platform) providing data about actual food choices on a gram-level. Due to this, the differences in the amounts of meat dish and salad and the shares of the meat dish in the total meal weights were able to be compared between the treatments. 

In this study, the vegetarian and meat-based dishes were identical (Supplementary File S2) except for the protein source; therefore, potential differences in appearance, e.g., color or scent were very unlikely to bias the results. The participants were given an opportunity to try both of the main dishes instead of only one of them in case they were completely unfamiliar with Härkis^®^ and, therefore, potentially more likely opt for the meat version. 

### 4.5. Limitations

The main limitation of the study was that due to the difficulties in the recruitment process caused by the COVID-19 pandemic, stratified randomization in terms of gender, age and BMI could not be performed. However, there were no statistically significant differences in any of the background factors between the treatments except for in terms of employment status (Table 1). Since students or those who are unemployed might not be able to purchase relatively expensive novel plant-based protein products, they might not be as familiar with them or might try them only under the study conditions when the meal was free.

The participants were able to choose one or both of the main dishes, which is not always the case in Finnish staff or university canteens. Participants with food allergies and insensitivities were excluded from the study; therefore, the results cannot be generalized to the entire adult population whose diets contain meat. The study was also conducted in an urban setting; therefore, the results may not be applicable to rural settings.

It may be possible that some participants may have opted for red meat, as Härkis^®^ may be considered as a more processed product and it contains additives [26]. However, as there were no statistically significant differences in the share of those who had eaten Härkis^®^ before across the treatments (Table 1), the choice of plant-based protein source was unlikely to bias the results. However, offering another more well-known plant-based protein source (e.g., soy bits) could have resulted in a different result.

The data collection period was between 4 PM and 7 PM on the first study day; whereas in the three other study days, it was between 3:30 PM and 6:30 PM. According to the latest national dietary study, the peak periods for highest energy intake are between 11 AM and noon and at 5 PM [16]. The time of the day may have affected the size of the meal for some participants, but since on the first data collection day the first participants that arrived were assigned into T1, on the second day into T2 and so forth, the treatments were unlikely to be affected differentially,

### 4.6. Future Directions

The meat and vegetarian options offered in this study were identical (Supplementary File S2) aside from replacing the minced beef with Härkis^®^. Since MMC is a traditional Finnish food, participants may have been skeptical of choosing only the FBC, since they had the opportunity to choose something with which they were more familiar. Therefore, there may be a need to make tempting new recipes from the plant-based protein sources instead. The use of self-reported weight and height data may result in underestimating the participants’ BMI [35]; therefore, using real measurements might increase the validity of the data. Considering the background factors such as gender, age, and BMI in the randomization process would be likely to strengthen the study design. Differential levels of hunger at the start of the study may also be a factor to consider.

Future studies should aim for a greater sample size and investigate food choice motives such as price or environmental awareness. The longitudinal effects of DoD and SA should also be investigated.

## 5. Conclusions

Neither main dish SA nor DoD strategy had an effect on Finnish adults’ likelihood of choosing a main dish with a plant-based protein source instead of red meat or the share of meat dish in the total meal weight. Results did not differ by gender, age, or BMI. More radical changes may have to be made in the food sequence in order to reduce meat consumption. Men were less likely to choose the vegetarian dish and had a larger share of the meat dish in their meal’s weight compared to women. A non-significant trend of those with higher BMI being more likely to choose the red meat version compared to those with normal weight was observed. The use of self-reported weight and height data may have led to the underestimation of the participants’ BMIs; therefore, real measurements and a larger sample size are needed in order to draw solid conclusions.

## Figures and Tables

**Table 1:** Characteristics of the participants (n = 163) in each treatment (T).

	T1	T2	T3	T4	Share	*p*-Value
n	41	42	40	40	-	-
Menu	Standard	DoC	Standard	DoD	-	-
Order of dishes	MMC first	MMC first	FBC first	FBC first	-	-
Women	51% (n = 21)	69% (n = 29)	63% (n = 25)	55% (n = 22)	59.50%	0.43 ^a^
Men	46% (n = 19)	31% (n = 13)	35% (n = 14)	43% (n = 17)	38.70%	
Other	0% (n = 0)	0% (n = 0)	3% (n = 1)	3% (n = 1)	1.20%	-
NA	2% (n = 1)	0% (n = 0)	0% (n = 0)	0% (n = 0)	0.60%	-
Age in years (mean, SD)	36.4 (13.0)	43.0 (13.3)	36.7 (12.8)	36.9 (14.4)	-	0.08 ^b^
BMI (mean, SD)	26.2 (6.4)	26.8 (5.7)	25.5 (4.6)	25.6 (5.2)	-	0.59 ^c^
Had tried Härkis^®^ before	85.0% (n = 34)	81.0% (n = 34)	90.0% (n = 36)	90.0% (n = 36)	-	0.63 ^a^
**Employment status**						
Employed	52.5% (n = 21)	66.7% (n = 28)	60.0% (n = 24)	47.5% (n = 19)	56.20%	0.048 ^a^
Student	35.0% (n = 14)	16.7% (n = 7)	40.0% (n = 16)	45.0% (n = 18)	34.00%	
Unemployed	10.0% (n = 4)	9.5% (n = 4)	0.0% (n = 0)	5.0% (n = 2)	6.20%	
Other	2.5% (n = 1)	7.1% (n = 3)	0.0% (n = 0)	2.5% (n = 1)	3.10%	
**Frequency of main meal of the day containing plant-based proteins**						
Every day	0.0% (n = 0)	0.0% (n = 0)	5.0% (n = 2)	7.5% (n = 3)	3.10%	0.51 ^a^
4–6 days per week	12.5% (n = 5)	7.1% (n = 3)	12.5% (n = 5)	12.5% (n = 5)	11.00%	
1–3 days per week	30.0% (n = 12)	38.1% (n = 16)	25.0% (n = 10)	35.0% (n = 14)	31.90%	
<1 day per week	57.5% (n = 23)	52.4% (n = 22)	50.0% (n = 20)	42.5% (n = 17)	50.30%	
Not sure	0.0% (n = 0)	2.4% (n = 1)	7.5% (n = 3)	2.5% (n = 1)	3.10%	
**Frequency of main meal of the day containing meat**						
Every day	22.5% (n = 9)	11.9% (n = 5)	20.0% (n = 8)	15.0% (n = 6)	17.20%	0.79 ^a^
4-6 days per week	42.5% (n = 19)	61.9% (n = 26)	50.0% (n = 20)	42.5% (n = 19)	51.50%	
1-3 days per week	25.0% (n = 10)	16.7% (n = 7)	27.5% (n = 11)	30.0% (n = 12)	24.50%	
<1 day per week	5.0% (n = 2)	7.1% (n = 3)	2.5% (n = 1)	7.5% (n = 3)	5.50%	
Not sure	0.0% (n = 0)	2.4% (n = 1)	0.0% (n = 0)	0.0% (n = 0)	0.60%	
**Frequency of trying new ingredients**						
Often	37.5% (n = 15)	35.7% (n = 15)	25.0% (n = 10)	40.0% (n = 16)	34.40%	0.87 ^a^
Sometimes	55.0% (n = 22)	54.8% (n = 23)	65.0% (n = 26)	52.5% (n = 21)	56.40%	
Rarely	7.5% (n = 3)	9.5% (n = 4)	10.0% (n = 4)	7.5% (n = 3)	8.60%	
Never	0.0% (n = 0)	0.0% (n = 0)	0.0% (n = 0)	0.0% (n = 0)	0.00%	

a = Fisher’s Exact Test, b = Kruskal–Wallis Test, c = one-way analysis of variance. BMI = body mass index; FBC = fava bean casserole; MMC = minced meat casserole; NA = not available; SD = standard deviation; T = treatment.

**Table 2:** The impact of Dish of the Day menu and sequence alteration in food selection by gender including only men and women. The “All”-category (n = 163) includes one participant in T1 who was unable to complete the questionnaire and two participants (one in T3 and one in T4) who reported their gender as “Other”.

		T1	T2	T3	T4	Share
Menu	-	Standard	DoD	Standard	DoD	-
Order of main dishes	-	MMC first	MMC first	FBC first	FBC first	-
n	All	41	42	40	40	100.0% (n = 163)
	Women	21	29	25	22	59.5% (n = 97)
	Men	19	13	14	17	38.7% (n = 63)
Chose FBC	All	17.1% (n = 7)	35.7% (n = 15)	17.5% (n = 7)	15.0% (n = 6)	21.4% (n = 35)
	Women	28.6% (n = 6)	37.9% (n = 11)	24.0% (n = 6)	22.7% (n = 5)	28.9% (n = 28)
	Men	5.3% (n = 1)	30.8% (n = 4)	0.0% (n = 0)	5.9% (n = 1)	9.5% (n = 6)
Chose both	All	36.6% (n = 15)	26.2% (n = 11)	37.5% (n = 15)	67.5% (n = 27)	41.7% (n = 68)
	Women	33.3% (n = 7)	27.6% (n = 8)	40.0% (n = 10)	63.6% (n = 14)	40.2% (n = 39)
	Men	36.8% (n = 7)	23.1% (n = 3)	35.7% (n = 5)	70.6% (n = 12)	42.6% (n = 27)
Chose MMC	All	46.3% (n = 19)	38.1% (n = 16)	45.0% (n = 18)	17.5% (n = 7)	36.8% (n = 60)
	Women	38.1% (n = 8)	34.5% (n = 10)	36.0% (n = 9)	13.6% (n = 3)	30.9% (n = 30)
	Men	57.9% (n = 11)	46.2% (n = 6)	64.3% (n = 9)	23.5% (n = 4)	47.6% (n = 30)
Mean meal weight in grams (SD)	All	441 (145)	390 (94)	406 (125)	448 (175)	-
	Women	373 (104)	371 (90)	355 (89)	386 (118)	-
	Men	522 (147)	433 (92)	482 (139)	530 (209)	-
Mean share of salad in the total meal weight (SD)	All	0.31 (0.11)	0.35 (0.09)	0.32 (0.11)	0.31 (0.10)	-
	Women	0.38 (0.11)	0.35 (0.10)	0.31 (0.07)	0.33 (0.09)	-
	Men	0.25 (0.07)	0.34 (0.09)	0.32 (0.17)	0.28 (0.11)	-
Mean share of FBC in the total meal weight (SD)	All	0.21 (0.26)	0.29 (0.28)	0.24 (0.26)	0.30 (0.20)	-
	Women	0.27 (0.30)	0.31 (0.28)	0.30 (0.28)	0.35 (0.21)	-
	Men	0.15 (0.21)	0.26 (0.27)	0.09 (0.14)	0.23 (0.16)	-
Mean share of MMC in the meal weight (SD)	All	0.47 (0.27)	0.36 (0.30)	0.45 (0.28)	0.39 (0.22)	-
	Women	0.35 (0.26)	0.34 (0.30)	0.39 (0.28)	0.32 (0.22)	-
	Men	0.61 (0.22)	0.40 (0.30)	0.58 (0.23)	0.49 (0.19)	-

DoD = Dish of the Day; FBC = fava bean casserole; MMC = minced meat casserole; SD = standard deviation; T = treatment.

**Table 3:** Main dish choices by treatment, gender, body mass index (BMI) group, and age group. Analyses were conducted using ordinal logistic regression.

	Choice			Crude Model	Multivariable Model
	Chose only FBC (%)	Chose both Main Dishes (%)	Chose only MMC (%)	*p*-Value	*p*-Value
**Treatment**					
T1	17.5% (n = 7)	35.0% (n = 14)	47.5% (n = 19)	0.13	0.23
T2	35.7% (n = 15)	26.2% (n = 11)	38.1% (n = 16)		
T3	15.4% (n = 6)	38.5% (n = 15)	46.2% (n = 18)		
T4	15.4% (n = 6)	66.7% (n = 26)	18.0% (n = 7)		
**Gender**					
Women	28.9% (n = 28)	40.2% (n = 39)	30.9% (n = 30)	0.004	0.002
Men	9.5% (n = 6)	42.9% (n = 27)	47.6% (n = 30)		
**BMI Group**					
18.5–24.9	25.0% (n = 20)	41.3% (n = 33)	33.8% (n = 27)	0.09	0.056
25–29.9	22.9% (n = 11)	41.7% (n = 20)	35.4% (n = 17)		
>30	6.5% (n = 2)	41.9% (n = 13)	51.6% (n = 16)		
**Age group**					
18–29	24.1% (n = 14)	34.5% (n = 20)	41.4% (n = 24)	0.70	0.63
30–44	27.1% (n = 13)	39.6% (n = 19)	33.3% (n = 16)		
45–65	14.3% (n = 8)	50.0% (n = 28)	35.7% (n = 20)		

BMI = body-mass index; FBC = fava bean casserole; MMC = minced meat casserole; SD = standard deviation; T = treatment.

**Table 4:** Share of the minced meat casserole (MMC) in the total meal weight by treatment, gender, body mass index (BMI) group, and age group. Analyses were conducted using linear models.

	Share of MMC in the Total Meal Weight (95%CI)	Crude Model	Multivariable Model
		*p*-Value	*p*-Value
**Treatment**			
T1	0.47 (0.39, 0.55)	0.18	0.33
T2	0.36 (0.28, 0.44)		
T3	0.46 (0.37, 0.54)		
T4	0.39 (0.31, 0.48)		
**Gender**			
Men	0.35 (0.30, 0.40)	<0.0001	<0.0001
Women	0.53 (0.46, 0.59)		
**BMI group**			
18.5–24.9	0.40 (0.34, 0.46)	0.25	0.11
25–29.9	0.42 (0.34, 0.50)		
>30	0.49 (0.40, 0.59)		
**Age group**			
18–29	0.44 (0.36, 0.51)	0.69	0.70
30–44	0.39 (0.31, 0.47)		
45–65	0.43 (0.36, 0.50)		

BMI = body-mass index; T = treatment.

## Data Availability

The data presented in this study are available on request from the corresponding author. The data are not publicly available due to it containing personal data from human subjects.

## Supplementary Materials

The following supporting information can be downloaded at: https://www.mdpi.com/article/10.3390/nu14193973/s1, File S1: Online Registration Form; File S2: Photography of Main Dishes.

## Abbreviations

DoD = Dish of the Day, FBC = fava bean casserole, MMC = minced meat casserole, NA = not available, SA = sequence alteration, SD = standard deviation, T = treatment, UTU = University of Turku.
