# Supplementary material for: Nudging Finnish Adults into Replacing Red Meat with Plant-Based Protein via Presenting Foods as Dish of the Day and Altering the Dish Sequence
Source: Nutrients. 2022 Sep 24;14(19):3973. doi: 10.3390/nu14193973 (PMC9573669; doi:10.3390/nu14193973)
Supplement: Supplementary file 1 [file nutrients-14-03973-s001.zip › Supplementary File S1.pdf]

ILMOITTAUDU MUKAAN RUOKATUTKIMUKSEEN

Pakolliset kysymykset merkitty tähdellä (*)

Tutkimukseen voi osallistua vain suomeksi.

Tämän tutkimuksen tavoitteena on tutkia suomalaisten ruokavalintoja ja ruokahävikkiä Flavoria®-tutkimusravintolassa. Etsimme tutkimukseemme 240 vapaaehtoista **18-65-vuotiasta, joiden ruokavalioon kuuluu liha ja joilla ei ole ruoka-aineallergioita tai -intoleransseja.** Ruokavalintasi tallennetaan vaakateknologian ja valokuvien avulla (sinua ei kuvata).

Tutkimukseen **osallistuminen kestää noin 45 minuuttia**, mutta todellinen kesto riippuu siitä, kuinka nopeasti sy annoksesi. Osallistua voit **joko keskiviikkona 8.12.2021, tiistaina 14.12.2021 tai keskiviikkona 15.12.2021 klo 15.30 - 18.30** välisenä aikana. **Pilottitutkimukseen 27.10.2021 osallistuneet eivät valitettavasti voi osallistua tähän tutkimukseen.**

Valittuasi buffetlinjastolta ateriasi voit rauhassa ruokailla ravintolassa muiden osallistujien kanssa. Sinua pyydetään vastaamaan lyhyeen kyselyyn aterian nauttimisen jälkeen.

Tutkimuskäynnillä **tarjottava ateria on ilmainen, ja siihen kuuluu jälkiruokakahvi/-tee.**

Teemme kaikkemme koronariskin minimoimiseksi ja osallistujan terveyden suojelemiseksi ja tästä syystä pidämme huolta turvajärjestelyistä Turun yliopiston ohjeistuksen mukaisesti. Kasvomaskeja ja käsidesiä on saatavilla tutkimustilanteessa.

Tulethan paikalle vain terveenä.

Lisätietoja tutkimuksesta:
Projektitutkija Esa-Pekka Nykänen
esa-pekka.nykanen@utu.fi
045-3205285

1. Hyväksyntä *

Olen tutustunut oheiseen tietosuojaselosteeseen
ja hyväksyn, että tutkimuksessa antamiani tietoja voidaan
käyttää tutkimustarkoitukseen selosteen kuvaamalla tavalla. Voin keskeyttää osallistumiseni tutkimukse
en
missä vaiheessa tahansa ilman, että tästä koituu minulle haittaa. Ilmoittautumisvaiheessa kerättyjä yhteystietoja ei yhdistetä tutkimusaineistoon ja ne hävitetään, kun tutkimus on ohi.

◯ Hyväksyn, että tutkimukseen osallistuessani vastauksiani käytetään tutkimustarkoituksessa seloste

◯ En hyväksy vastausteni käyttöä

2. Oletko 18-65-vuotias? *

- ◯ Kyllä
- ◯ En

3. Kuuluuko liha (sika/nauta/kana/muu siipikarja) ruokavalioosi? *

- ◯ Kyllä
- ◯ Ei

4. Onko sinulla ruoka-aineallergioita ja/tai -intoleransseja?
(esim. maito, viljat, gluteeni, palkokasvit, sipuli, kananmuna, pähkinät, siemenet) *

- ◯ Kyllä
- ◯ Ei

5. Osallistuitko pilottitutkimukseen Flavorialla 27.10.2021 klo 16-19?

- ◯ Kyllä
- ◯ En

6. Valitse alla olevista ajoista itsellesi sopiva tutkimusaika. *

- ◯ ke 8.12. klo 15.30 - 16.00
- ◯ ke 8.12. klo 16.00 - 16.30
- ◯ ke 8.12. klo 16.30 - 17.00
- ◯ ke 8.12. klo 17.00 - 17.30
- ◯ ke 8.12. klo 17.30 - 18.00
- ◯ ke 8.12. klo 18.00 - 18.30
- ◯ ti 14.12. klo 15.30 - 16.00
- ◯ ti 14.12. klo 16.00 - 16.30
- ◯ ti 14.12. klo 16.30 - 17.00
- ◯ ti 14.12. klo 17.00 - 17.30
- ◯ ti 14.12. klo 17.30 - 18.00

◯ ti 14.12. klo 18.00 - 18.30

◯ ke 15.12. klo 15.30 - 16.00

◯ ke 15.12. klo 16.00 - 16.30

◯ ke 15.12. klo 16.30 - 17.00

◯ ke 15.12. klo 17.00 - 17.30

◯ ke 15.12. klo 17.30 - 18.00

◯ ke 15.12. klo 18.00 - 18.30

7. Kerro yhteystietosi.

Etunimi *	
Sukunimi *	
Matkapuhelin	
Sähköposti *	
